# Molecular epidemiology of *Acinetobacter baumannii* during COVID-19 at a hospital in northern China

**DOI:** 10.1186/s12941-024-00716-0

**Published:** 2024-07-18

**Authors:** Xinlin Huang, Nianzhi Ning, Deyu Li, Suming Chen, Liangyan Zhang, Huan Wang, Chunmei Bao, Xiaolan Yang, Boan Li, Hui Wang

**Affiliations:** 1 State Key Laboratory of Pathogen and Biosecurity, Beijing Institute of Microbiology and Epidemiology, No. 20 Dongda Street, Fengtai District, Beijing, 100071 China; 2 Department of Clinical Laboratory, the Fifth Medical Center, Chinese Peoples’s Liberation Army (PLA) General Hospital, No. 100 Western 4th Middle Ring Road, Beijing, 100039 China; 3 School of Medical Laboratory, Weifang Medical University, Weifang, 261053 China

**Keywords:** *Acinetobacter baumannii*, Molecular epidemiology, Resistance mechanisms, ST540, Tn*2009*

## Abstract

**Background:**

The wide spread of carbapenem-resistance clones of *Acinetobacter baumannii* has made it a global public problem. Some studies have shown that the prevalence of *Acinetobacter baumannii* clones can change over time. However, few studies with respect to the change of epidemiological clones in *Acinetobacter baumannii* during Corona Virus Disease 2019 (COVID-19) were reported. This study aims to investigate the molecular epidemiology and resistance mechanisms of *Acinetobacter baumannii* during COVID-19.

**Results:**

A total of 95 non-replicated *Acinetobacter baumannii* isolates were enrolled in this study, of which 60.0% (*n* = 57) were identified as carbapenem-resistant *Acinetobacter baumannii* (CRAB). The positive rate of the *bla*_*OXA−23*_ gene in CRAB isolates was 100%. A total of 28 Oxford sequence types (STs) were identified, of which the most prevalent STs were ST540 (*n* = 13, 13.7%), ST469 (*n* = 13, 13.7%), ST373 (*n* = 8, 8.4%), ST938 (*n* = 7, 7.4%) and ST208 (*n* = 6, 6.3%). Differently, the most widespread clone of *Acinetobacter baumannii* in China during COVID-19 was ST208 (22.1%). Further study of multidrug-resistant ST540 showed that all of them were carrying *bla*_*OXA−23*_, *bla*_*OXA−66*_, *bla*_*ADC−25*_ and *bla*_*TEM−1D*_, simultaneously, and first detected Tn*2009* in ST540. The *bla*_*OXA−23*_ gene was located on transposons Tn*2006* or Tn*2009*. In addition, the ST540 strain also contains a drug-resistant plasmid with *msr(E)*, *armA*, *sul1* and *mph(E)* genes.

**Conclusion:**

The prevalent clones of *Acinetobacter baumannii* in our organization have changed during COVID-19, which was different from that of China. ST540 strains which carried multiple drug-resistant mobile elements was spreading, indicating that it is essential to strengthen the molecular epidemiology of *Acinetobacter baumannii*.

## Introduction

*Acinetobacter baumannii (A.baumannii)* is a common opportunistic pathogen causing infections, including hospital-acquired pneumonia, bacteremia, urinary tract infections, and traumatic injuries [1]. Multiple intrinsic and acquired resistance mechanisms [2]have made multidrug-resistant (MDR), extensively drug-resistant (XDR), and pan-drug-resistant (PDR) *A. baumannii* strains increasingly common and made treatment increasingly harder [3–5]. Due to its high rate of resistance to carbapenems, the list of bacterial pathogens in urgent need of novel antibiotics issued by the World Health Organization (WHO) ranked carbapenem-resistant *A.baumannii* as the highest priority in 2017 [6].

β-lactamases are the main reason for bacterial resistance to carbapenems and could be classified into class A, class B, class C and class D [7]. Carbapenems belong to class D β-lactamases, which consist of *bla*_*OXA−23−like*_, *bla*_*OXA−40−like*_, *bla*_*OXA−51−like*_, *bla*_*OXA−58−like*_, and *bla*_*OXA−23*_. They are the leading cause of carbapenem resistance in *A. baumannii* [8]. Some studies have shown that the *bla*_OXA−23_ gene is the most prevalent class D β-lactamases gene [9] and can be inserted into the plasmid or chromosome so that *A. baumannii* can acquire drug resistance quickly [10]. The *bla*_OXA−23_ gene is usually located in transposons Tn*2006*, Tn*2007*, Tn*2008*, and Tn*2009*. Tn*2006* is the most common type of transposons worldwide, while Tn*2009* is mainly popular in China [11]. In addition, plasmids carrying resistance genes can help *A.baumannii* reduce antibiotic susceptibility and better adapt to the environment [12].

To better elucidate the molecular epidemiology of pathogens, Maiden et al. invented the Multilocus Sequence Typing (MLST) method in 1998 [13]. The MLST has now been applied in molecular epidemiology for disease transmission and virulence evolution, surveillance in public health, and many other fields [14–16]. According to the results of epidemiological studies on *A. baumannii*, CRAB has increased globally due to the worldwide prevalence of Global Clone 1 (GC1) and Global Clone 2 (GC2) [9]. CRAB has different mainstream STs in different regions. ST191, ST195, and ST208 are common STs in China [17, 18], and all three STs originated from GC2. The main prevalent clone in Italy is ST78 [19], and in central Greece, ST101 [20], while in the United States, ST208 and ST281 are predominant [21].

Studies have shown that drug resistance in *A.baumannii* is associated with predominant STs [22], and the predominant ST type of *A.baumannii* in the same region could be replaced by others with a higher growth rate [23]. Previously, we conducted molecular epidemiological studies on *A.baumannii* collected over a long period in a Beijing hospital. In this study, we found ST191 was replaced by other ST and demonstrated an outbreak of infection with ST195 carrying the *bla*_*OXA−23*_ resistance gene in northern China for the first time [24]. To our knowledge, there have been few studies on the long-term epidemic evolution of *A.baumannii* in a single medical machine, especially after the outbreak of COVID-19. Therefore, this study aimed to investigate the molecular epidemiology and resistance mechanisms of *Acinetobacter baumannii* in a single institution. The findings of this work will provide important insights for controlling the spread of CRAB and minimizing the incidence of untreatable infections in clinical settings.

## Methods

### Strain and identification

From January 2020 to December 2022, 95 strains of *A.baumannii* were collected from a single hospital in Beijing, China. Only the first isolate from each patient was included in the study. The samples were obtained from sputum, abdominal/pleural fluid, blood, urine, throats, and so on, all of which were initially identified as *Acinetobacter spp.* by the VITEK-2 system (BioMérieux France) and later identified to the species level by Microflex LT Time-of-Flight Mass Spectrometer.

### Antimicrobial susceptibility tests

All *A. baumannii* strains were tested for susceptibilities to 15 antibiotics, including ticarcillin/clavulanic acid (TCC), piperacillin/tazobactam (TZP), ceftazidime (CAZ), cefoperazone/sulbactam (CFP), cefepime (FEP), imipenem (IPM), meropenem (MEM), tobramycin (TOB), ciprofloxacin (CIP), levofloxacin (LVX), tigecycline (TGC), colistin (COL), minocycline (MNO), doxycycline (DOX) and trimethoprim/sulfamethoxazole (TMP), using a VITEK-2 compact system with AST-N-335 cards. The results were evaluated according to the Clinical and Laboratory Standards Institute (CLSI) criteria (M100-S32).

### Genome sequencing

Takara DNA extraction kit was used to extract the genomic DNA of the 95 strains. All strains were whole genome sequenced using the Illumina Hiseq X10 platform, with the 2*150 bp paired-end sequencing strategy. The genome was assembled and annotated using SPAdes [25] and Prokka [26].

### Phylogenetic analysis

Single-nucleotide polymorphisms (SNPs) were extracted using Snippy (https://github.com/tseemann/snippy) to generate core genomic alignment. The core genomic alignment was used to generate a maximum likelihood (ML) phylogenetic tree by using Fasttree2 [27] (MDR-ZJ06 [28] as a reference sequence). And the results were shown by iTOL [29].

### Multilocus sequence typing (MLST)

MLST analyses were performed using the Oxford and Pasteur scheme. The sequence types (STs) and allelic profiles were analyzed using the PubMLST database (http://pubmlst.org/abaumannii/ ). The newly identified STs were submitted to the MLST database curator for approval, and an ST number was assigned. A minimum-spanning tree based on the allelic difference between isolates of the seven housekeeping genes was constructed using PHYLOViZ Online [30].

### Identification of resistance genes and drug-resistant mobile elements

All the antimicrobial resistance genes were identified using the Resfinder [31] software. We take the assembled contigs of ST540 genomes to the VRprofile2 pipeline [32] (https://tool2-mml.sjtu.edu.cn/VRprofile/) to classify contigs as the fragments of chromosomes or plasmids and to identify whether they carried resistance genes. To further study the plasmids with resistance genes, we used blastn to compare these contigs to the NCBI RefSeq plasmids database (https://ftp.ncbi.nlm.nih.gov/refseq/release/plasmid/) to find the reference sequence. For the drug-resistant plasmid of ST540, we use p2BJAB07104 (NC_021728.1) with 84% coverage and 100% identity as reference sequences to study the plasmid structure. Mauve [33] was used to identify all the contigs located in the resistant plasmid, by comparing strain contigs with the reference genome BJAB07104 (NC_021726.1) which contains the plasmid p2BJAB07104 (NC_021728.1). Plasmid maps were presented using BRIG [34]. To determine whether the contig carrying *bla*_*OXA−23*_ gene contains insertion sequences, we submitted it to ISfinder [35](http://www-is.biotoul.fr). To further identified the type of transposon of contigs carrying the *bla*_*OXA−23*_ gene, we compared these contigs with transposon reference sequences (Tn*2006* EF127491.1, Tn*2007* EF059914.1, Tn*2008* KP074966.1, Tn*2009* CP097879.1) using BLAST (ncbi-blast-2.11.0).

## Results

### Molecular epidemiology

**Fig. 1:**
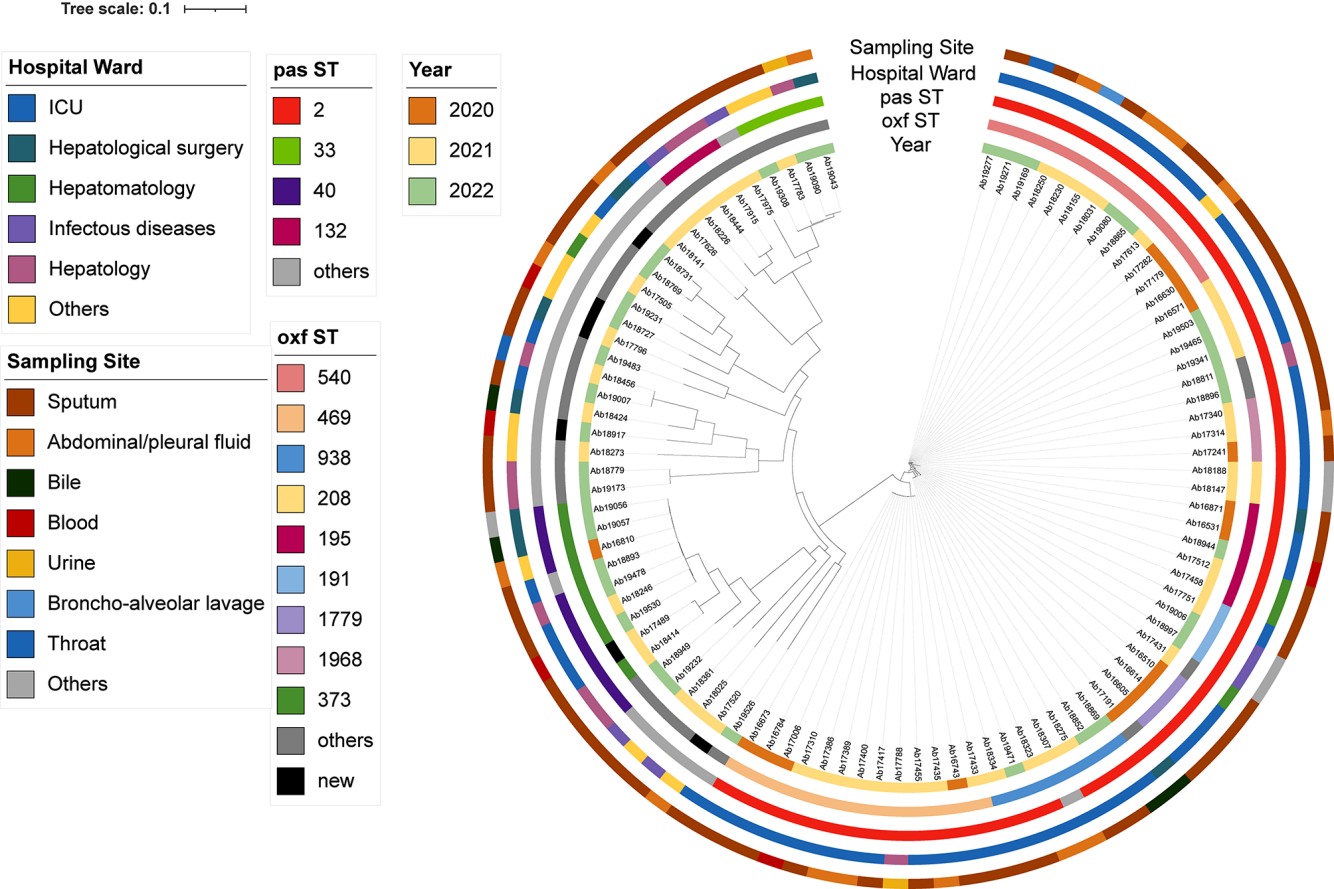
Evolutionary relationship of 95 strains of *Acinetobacter baumannii*. The evolutionary relationships of the 95 *Acinetobacter baumannii* strains were represented using an evolutionary tree, with the circles from inner to outer being: year of isolation, oxf ST, pas ST, hospital ward, and sampling site

**Fig. 2:**
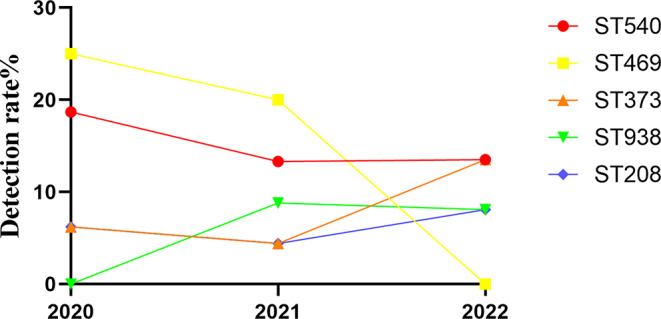
Top 5 ST-type detection rate. The horizontal and vertical coordinates represent the detection rate and the year of detection

A total of 95 non-repeating *A.baumannii* strains collected from January 2020 to December 2022 were enrolled in this study. The result of the MSLT Pasteur scheme showed that a total of 21 STs were found and the prevalent ST was ST2 (*n* = 56, 58.9%), ST40 (*n* = 9, 9.5%), and ST33 (*n* = 4, 4.2%). The Oxford scheme identified 28 STs, the most prevalent STs were ST540 (*n* = 13, 13.7%), ST469 (*n* = 13, 13.7%), ST373 (*n* = 8, 8.4%), ST938 (*n* = 7, 7.4%) and ST208 (*n* = 6, 6.3%). In this study, more than half of *A. baumannii* came from ICU (*n* = 56, 58.9%), followed by hepatological surgery (*n* = 17, 17.9%). The most common source of samples was sputum (*n* = 73, 65.7%) Fig. 1. As shown in Fig. 2, the detection rate of ST540 was equivalent to that of ST469. However, we could not find any strain belonging to ST469 in 2022.

**Fig. 3:**
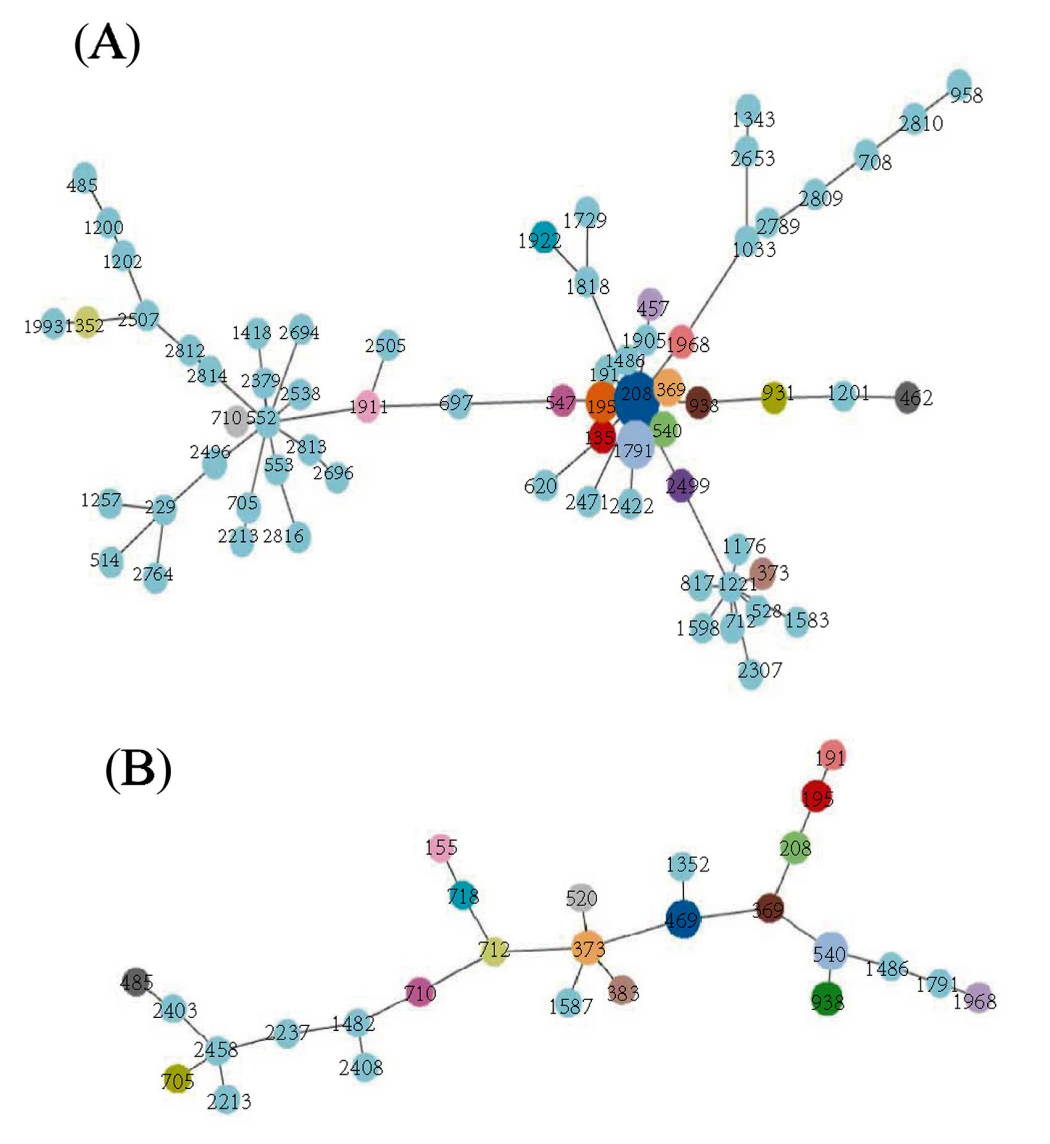
ST-type minimum-spanning tree. (**A**) Genetic relationship of most *Acinetobacter baumannii* isolates of China. (**B**) genetic relationship of *Acinetobacter baumannii* isolates of this study. The size of the dots is proportional to the number of strains

To investigate the clonal evolution of *A.baumannii* during COVID-19, we studied the molecular epidemiology of *A. baumannii* in China (published on the NCBI website) from 2020 to 2022 and then performed a minimum-spanning tree analysis. The results showed that ST208(*n* = 68, 22.1%) was the most prevalent clone in China during this period, which is different from our findings (Fig. 3).

### Antimicrobial susceptibility tests

**Fig. 4:**
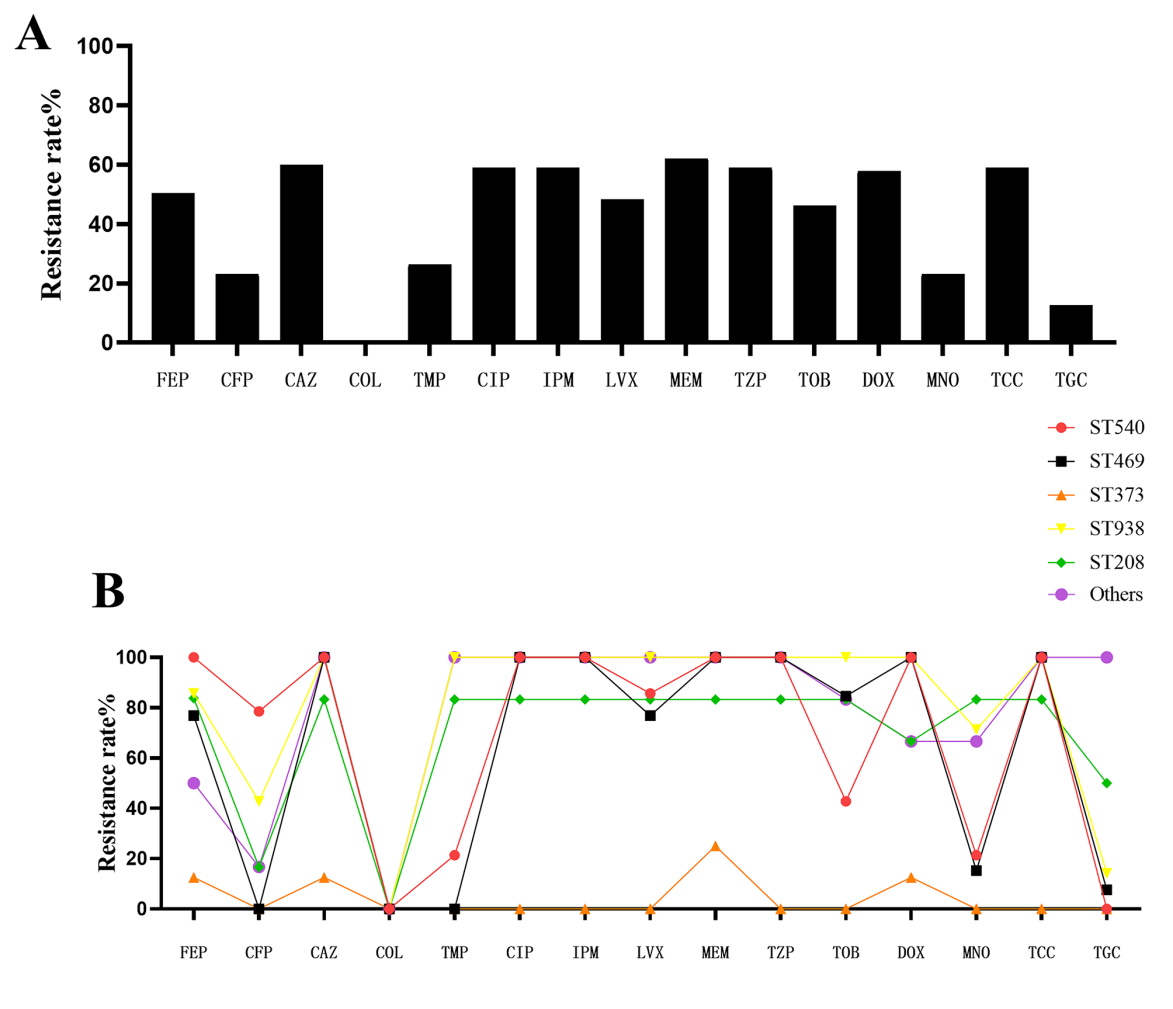
Rates of antimicrobial resistance among isolates. (**A**)Shows the 95 strains of *Acinetobacter baumannii* ‘s overall antimicrobial resistance rate, and (**B**) shows the resistance rate of the top 5 ST types of detection rate

The minimal inhibitory concentration (MIC) values against 15 kinds of drugs were determined for all *A. baumannii*. Figure 4 A shows the drug resistance rate of 95 *A. baumannii* strains. The results showed that most *A. baumannii* strains were carbapenem-resistant *A. baumannii* (CRAB), with 58.9% against imipenem and 62.1% against meropenem. All isolates were susceptible to colistin, and 12 (12.6%) strains were resistant to tigecycline. Figure 4 B shows the resistance rates of the top 5 STs of strains. The antibiotic resistant rates of ST540, ST469, ST 938 and ST208 against carbapenems were above 80%, but all ST373 strains were sensitive to carbapenems. ST540 has the highest resistance rate to cefoperazone/sulbactam, reaching 78.5%.

### Drug-resistance genes

**Fig. 5:**
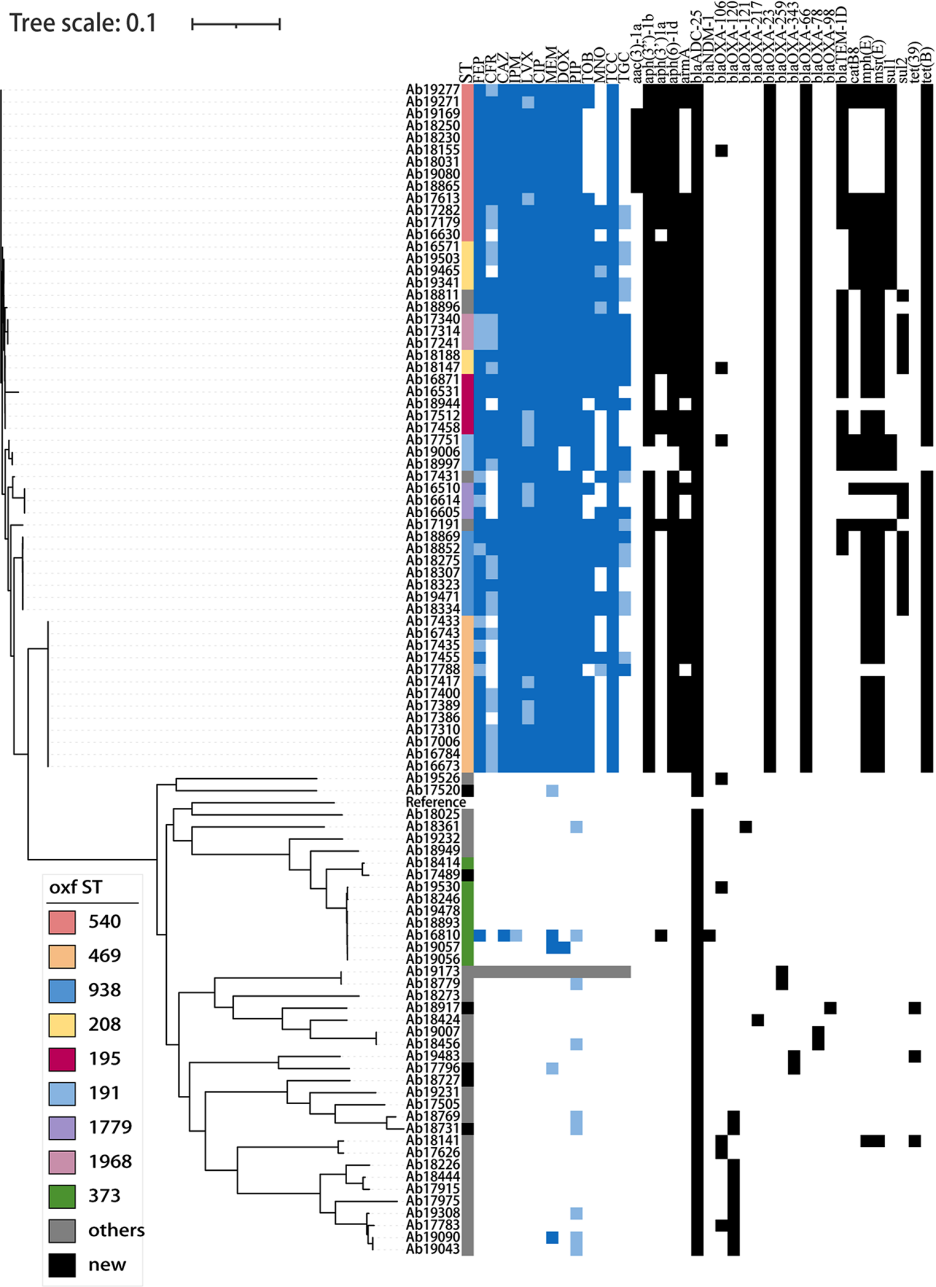
Heat map of antimicrobial resistance genes. Different colors in the legend on the left represent different oxf ST types. The blue portion of the heatmap indicates antimicrobial drug susceptibility testing, dark blue indicates resistance, light blue indicates intermediary, white indicates susceptibility, and gray indicates vacancy. The black portion of the heatmap represents resistance genes, black indicates the presence of the gene, and white indicates the absence of the gene

Genomic analysis showed that 95 *A. baumannii* contained a total of 30 resistance genes. The carbapenemase gene *bla*_*OXA−25*_ was present in all *A. baumannii*, and *bla*_*OXA−23*_ and *bla*_*OXA−66*_ were present in all CRAB isolates. The other resistance genes detected in our study included: carbapenem (*bla*_*NDM−1*_, *bla*_*OXA−106*_, *bla*_*OXA−120*_, *bla*_*OXA−121*_, *bla*_*OXA−217*_, *bla*_*OXA−23*_, *bla*_*OXA−259*_, *bla*_*OXA−343*_, *bla*_*OXA−374*_, *bla*_*OXA−377*_, *bla*_*OXA−430*_, *bla*_*OXA−51*_, *bla*_*OXA−66*_, *bla*_*OXA−78*_, *bla*_*OXA−98*_, *bla*_*TEM−1D*_), aminoglycoside (*aac* [3]*-Ia, aph(3’)-Ia, aph(3’ ‘)-Ib, aph(6’)-Id, aph(3’)-VI, armA*), macrolide (*mph(E), msr(E)*), *phenicol* (*catB8*), tetracycline (*tet(B)*) and sulfonamide (*sul1, sul2*) (Fig. 5). Interestingly, the ST540 strains contain a different resistance gene profile, some ST540 strains do not carry *catB8*, *mph(E), msr(E)* genes.

### Drug-resistant mobile elements

**Fig. 6:**
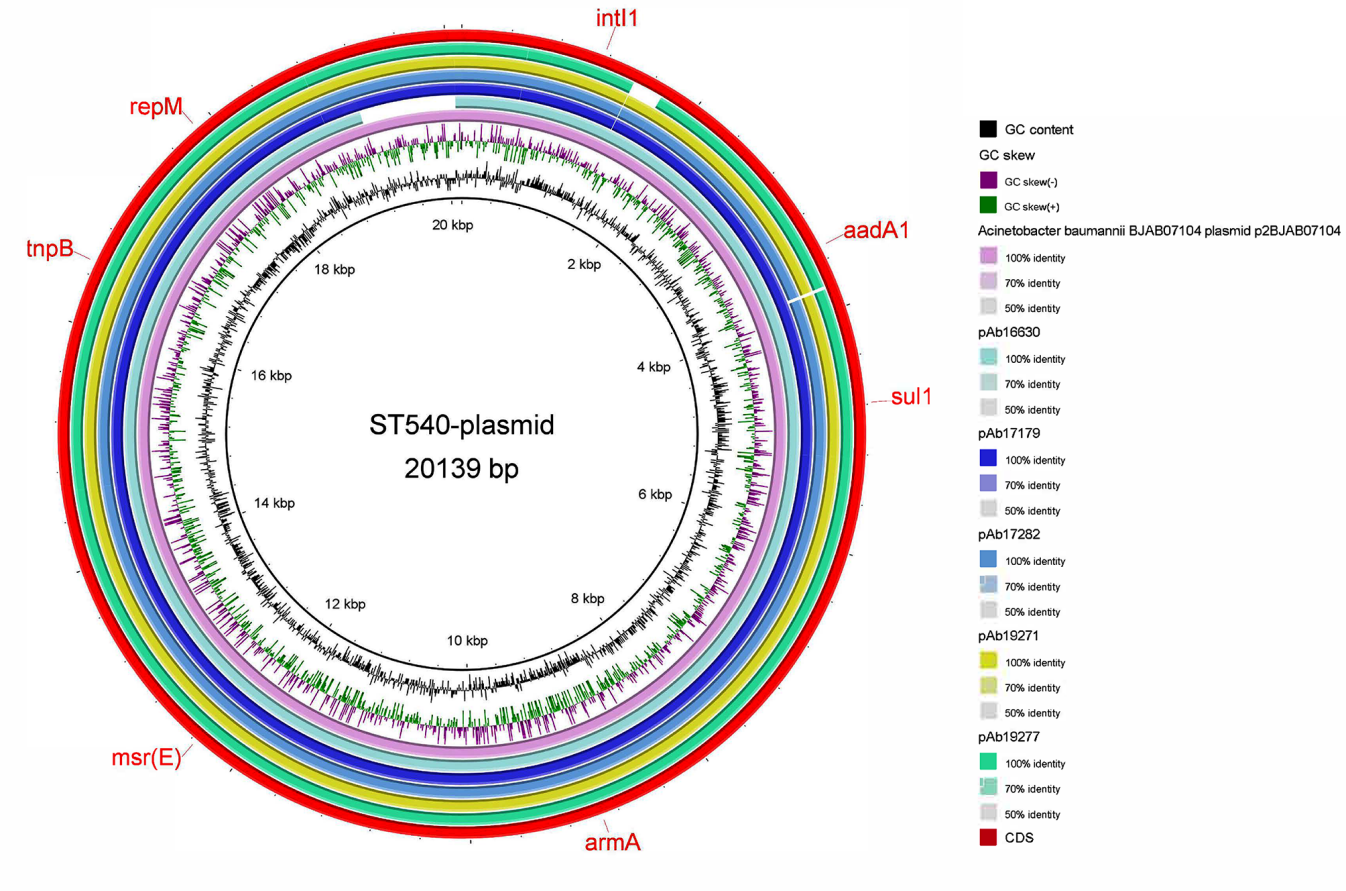
ST540 drug-resistant plasmids. Sequence comparison of plasmid p2BJAB07104 and other plasmids including pAb16630, pAb17179, pAb17282, pAb19271, pAb19277. BLASTN matches with an identity between 0 and 100% are colored in gradient

**Fig. 7:**
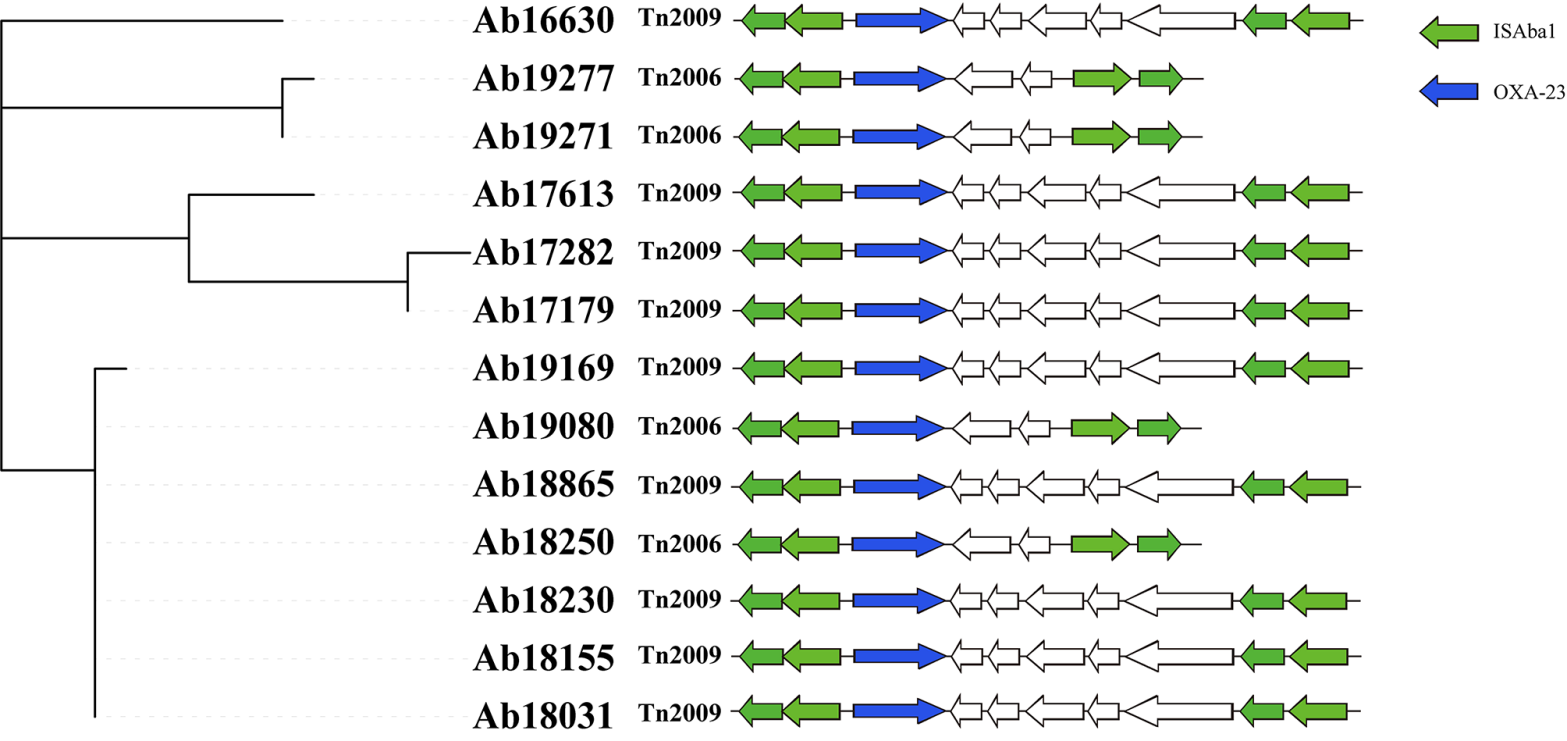
ST540 Transposons. The evolutionary relationships within ST540 are shown on the left, the transposons carried by each strain are shown on the right, and the direction of the arrow indicates the insertion sequence direction

We suspected that plasmid carrying was the cause of the difference in the ST540 resistance gene profile. Thus we uploaded ST540 strain contigs to the VRprofile2 pipeline to predict whether ST540 carried resistant plasmids. The results showed that five strains carried resistant plasmid Fig. 6. Analysis by blastn showed that these resistant plasmids were high similar to p2BJAB07104 (NC_021728.1) with 84% coverage and 100% identity. We spliced the plasmids and found that they all carried *msr(E), armA, sul1*, and *mph(E)* genes, which were not found on the chromosomes. None of the *bla*_*OXA−23*_ gene was located on the resistant plasmid. To study its genetic environment, we compared the ST540 strain contigs carrying *bla*_*OXA−23*_ gene with the transposon reference sequence (Tn*2006* EF127491.1, Tn*2007* EF059914.1, Tn*2008* KP074966.1, Tn*2009* CP097879.1) and found that the *bla*_*OXA−23*_ gene was located on Tn*2006* (*n* = 4, 30.8%) and Tn*2009* (*n* = 9, 69.2%%) Fig. 7. Tn*2006* and Tn*2009* share a common region, “OXA-23”. They also contain *ISAba1*. The two *ISAba1* copies were inversely orientated in Tn*2006* compared with being the same direction in Tn*2009*.

## Discussion

Due to its easy access to resistance genes, *A.baumannii* is considered a major threat to global public health [6]. In order to investigate the epidemical clones change and drug resistance mechanism of *A.baumannii* during COVID-19 and prevent the outbreak of infection caused by Multi-drug Resistant *Acinetobacter baumannii* (MDR-Ab), we retrospectively analyzed the molecular epidemiological characteristics and drug resistance mechanism of 95 *A.baumannii* strains at a hospital in Beijing for 3 years (2020–2022).

CHINET data showed that the resistance rate of *Acinetobacter baumannii* to carbapenem in China increased approximately twofold from 2005 (39%) to 2019 (79%) [36]. However, the resistance rate of carbapenems has decreased, from 79 to 71.9%, between 2019 and 2022 during the COVID-19 pandemic [36]. In this study, the resistance rate of carbapenem was only 62.1%, which was lower than the data (71.9%)in China. The variation of epidemic clones may be an important reason for the decrease in drug resistance rate. For example, there was a study reported that the variation of STs in a single hospital accompanied with the resistance rate to amikacin and tobramycin increased from 46.6 to 92% [37]. In this study, we found CRAB ST208 was still the main epidemic clone in China, reaching 22.1%, while in our hospital ST208 accounted for only 6.3%. In addition, we also found the sensitive *A. baumannii* ST373, which accounted for 8.4% of the total isolation rate. Thus, our study suggests that epidemiologic clonal changes may be important in influencing resistance rates. In China, the TGC resistance rate of *A. baumannii* was only 2% in 2022, which is much lower than other countries, for instance, Turkey, whose TGC resistance rate was about 81%, and Israel, 66%, Kuwait, 13.6% [36, 38, 39]. The treatment of MDR-Ab in our hospital usually involves tigecycline or colistin, as these two drugs have become the last line of defense in treating MDR-Abb [40]. In this study, the resistance rate of tigecycline is 12.6%, which is higher than the 4% rate in China [41], indicating a trend towards an increased level of tigecycline resistance. This trend is likely to pose a threat to the effectiveness of this last-resort drug. In order to prevent *A. baumannii* from becoming an incurable bug, the development of novel drugs is an urgent need. Our study provides additional bacterial data to support further investigation into therapeutic strategies for CRAB.

Our study found that ST540 became the main epidemic clone in our hospital, which was different from our previous report that ST195 and ST191 were the main STs in this hospital before 2014 [24]. Since the first report of ST540 in China in 2013 [42], it has increased in recent years in the northern China of Shandong Province [43] (12.8%) and Jilin Province [44](14.6%). Unlike these reports from China, we found for the first time that ST540 was the most prevalent clone in a single hospital. All ST540 strains isolated in this study were 100% resistant to carbapenems. CRAB is a worldwide problem because these strains are often resistant to all other commonly used antibiotics [45]. Although *A.baumannii* strains carrying *bla*_*OXA−23*_ and *bla*_*TEM−1D*_ have been sporadically reported [46], the coexistence of *bla*_*OXA−23*_ and *bla*_*TEM−1D*_ with other carbapenemase genes in *A. baumannii* is rarely reported. In this study, all ST540 strains carried *bla*_*OXA−23*_, *bla*_*OXA−66*_, *bla*_*ADC−25*_ and *bla*_*TEM−1D*_ genes, which may be the main cause of resistance to carbapenem. To our knowledge, this is the first report of a clinical *A. baumannii* ST540 isolates carrying *bla*_*OXA−23*_, *bla*_*OXA−66*_, *bla*_*ADC−25*_ and *bla*_*TEM−1D*_ genes in northern China. In addition, ST540 strains also carried resistance genes related to aminoglycoside, macrolide, phenicol, tetracycline, and sulfonamide. This finding is in agreement with previous observations reporting that most *A. baumannii* isolates carrying the *bla*_*OXA−23*_ gene also carry resistance genes to aminoglycosides and/or tetracyclines [45, 47].

*Acinetobacter baumannii* has the property of natural transformation, which can pass drug-resistant genes to other strains through the genome or mobile elements [48]. In our study, we also found internal differences in the resistance of ST540 due to the presence of [49]resistant plasmids carrying *msrE, armA, sul1* and *mphE* genes, causing these strains to be resistant to macrolide, aminoglycoside and sulfonamide, in only five strains of ST540. The plasmid was unclassified plasmid and first identified on MDR-ZJ06 strain in China in 2011 [42]. Now, this plasmid could be found in some of the ST540 strains, suggesting that it may spread in *A. baumannii* through horizontal gene transfer. The *bla*_*OXA−23*_ genes are usually carried by transposons such as Tn*2006*, Tn*2007*, Tn*2008*, and Tn*2009* [9]. Tn*2006* is the most prevalent transposon worldwide and the only one that has been tested to be able to move independently and could be fund in many different chromosomal and plasmid contexts in distantly related *A. baumannii* strains [7]. According to a survey, the Tn*2006*-possessing strains belonged to various STs, whereas Tn*2009*-possessing strains were mainly belonged to ST191 [50]. However, our study found for the first time in ST540 that 69.2% of the strains carried Tn*2009*, indicating that Tn*2009* is transferring among different *A. baumannii* strains.

## Conclusion

During the COVID-19 pandemic, we investigated *A. baumannii* in a hospital in northern China and observed a rise in resistance to TGC during this time. ST540, carrying the resistance plasmid and Tn2009/Tn2006, became the predominant clone in our study unit. Our genomic analysis can help track outbreaks, detect the evolution of prevalent clones, and identify the presence of resistance genes. All these massages can help optimize antimicrobial stewardship and provide essential information for treatment decision-making. At the same time, our findings offer new epidemiological data that can be used to enhance infection control measures and clinical treatments in China.

## Data Availability

The whole genome sequencing data in this study have been deposited in Genbank under BioProject ID PRJNA1005007.

## Abbreviations

A.baumannii: *Acinetobacter Baumannii*
COVID-19: Corona Virus Disease 2019
CRAB: Carbapenem-Resistant *Acinetobacter Baumannii*
STs: Sequence Types
MDR: Multidrug-Resistant
XDR: Extensively Drug-Resistant
PDR: Pan-Drug-Resistant
WHO: World Health Organization
MLST: Multilocus Sequence Typing
GC1: Global Clone 1
GC2: Global Clone 2
TCC: Ticarcillin/Clavulanic Acid
TZP: Piperacillin/Tazobactam
CAZ: Ceftazidime
CFP: Cefoperazone/Sulbactam
FEP: Cefepime
IPM: Imipenem
MEM: Meropenem
TOB: Tobramycin
CIP: Ciprofloxacin
LVX: Levofloxacin
TGC: Tigecycline
COL: Colistin
MNO: Minocycline
DOX: Doxycycline
TMP: Trimethoprim/Sulfamethoxazole
CLSI: Clinical and Laboratory Standards Institute
SNPs: Single-Nucleotide Polymorphisms
ML: Maximum Likelihood
MIC: Minimal Inhibitory Concentration
MDR-Ab: Multi-Drug Resistant *Acinetobacter baumannii*

## References

[1] Almasaudi SB. Acinetobacter spp. as nosocomial pathogens: epidemiology and resistance features. Saudi J Biol Sci. 2018;25(3):586–96. 29686523 10.1016/j.sjbs.2016.02.009 PMC5910652

[2] Lee CR, Lee JH, Park M, Park KS, Bae IK, Kim YB, et al. Biology of Acinetobacter baumannii: Pathogenesis, Antibiotic Resistance mechanisms, and prospective treatment options. Front Cell Infect Microbiol. 2017;7:55. 28348979 10.3389/fcimb.2017.00055 PMC5346588

[3] Paterson DL, Bonomo RA. Multidrug-resistant gram-negative pathogens: the Urgent need for ‘Old’ polymyxins. Adv Exp Med Biol. 2019;1145:9–13. 31364068 10.1007/978-3-030-16373-0_2

[4] Nasr P. Genetics, epidemiology, and clinical manifestations of multidrug-resistant Acinetobacter baumannii. J Hosp Infect. 2020;104(1):4–11. 31589900 10.1016/j.jhin.2019.09.021

[5] Li T, Luo D, Ning N, Liu X, Chen F, Zhang L, et al. Acinetobacter baumannii adaptation to the host pH microenvironment is mediated by allelic variation in a single residue of BauA protein. PNAS Nexus. 2023;2(4):pgad079. 37065616 10.1093/pnasnexus/pgad079 PMC10098034

[6] Tacconelli E, Carrara E, Savoldi A, Harbarth S, Mendelson M, Monnet DL, et al. Discovery, research, and development of new antibiotics: the WHO priority list of antibiotic-resistant bacteria and tuberculosis. Lancet Infect Dis. 2018;18(3):318–27. 29276051 10.1016/S1473-3099(17)30753-3

[7] Mugnier PD, Poirel L, Naas T, Nordmann P. Worldwide dissemination of the blaOXA-23 carbapenemase gene of Acinetobacter baumannii. Emerg Infect Dis. 2010;16(1):35–40. 20031040 10.3201/eid1601.090852 PMC2874364

[8] Huang ZY, Li J, Shui J, Wang HC, Hu YM, Zou MX. Co-existence of blaOXA-23 and blaVIM in carbapenem-resistant Acinetobacter baumannii isolates belonging to global complex 2 in a Chinese teaching hospital. Chin Med J (Engl). 2019;132(10):1166–72. 30882466 10.1097/CM9.0000000000000193 PMC6511418

[9] Hamidian M, Nigro SJ. Emergence, molecular mechanisms and global spread of carbapenem-resistant Acinetobacter baumannii. Microb Genom. 2019;5(10). 10.1099/mgen.0.000306 PMC6861865 31599224

[10] Thomas CM, Nielsen KM. Mechanisms of, and barriers to, horizontal gene transfer between bacteria. Nat Rev Microbiol. 2005;3(9):711–21. 16138099 10.1038/nrmicro1234

[11] Nigro SJ, Hall RM. Structure and context of Acinetobacter transposons carrying the oxa23 carbapenemase gene. J Antimicrob Chemother. 2016;71(5):1135–47. 26755496 10.1093/jac/dkv440

[12] Khongfak S, Thummeepak R, Leungtongkam U, Tasanapak K, Thanwisai A, Sitthisak S. Insights into mobile genetic elements and the role of conjugative plasmid in transferring aminoglycoside resistance in extensively drug-resistant Acinetobacter baumannii AB329. PeerJ. 2022;10:e13718. 35855908 10.7717/peerj.13718 PMC9288165

[13] Maiden MC, Bygraves JA, Feil E, Morelli G, Russell JE, Urwin R, et al. Multilocus sequence typing: a portable approach to the identification of clones within populations of pathogenic microorganisms. Proc Natl Acad Sci U S A. 1998;95(6):3140–5. 9501229 10.1073/pnas.95.6.3140 PMC19708

[14] Pérez-Losada M, Cabezas P, Castro-Nallar E, Crandall KA. Pathogen typing in the genomics era: MLST and the future of molecular epidemiology. Infect Genet Evol. 2013;16:38–53. 23357583 10.1016/j.meegid.2013.01.009

[15] Bessell PR, Rotariu O, Innocent GT, Smith-Palmer A, Strachan NJ, Forbes KJ, et al. Using sequence data to identify alternative routes and risk of infection: a case-study of campylobacter in Scotland. BMC Infect Dis. 2012;12:80. 22462563 10.1186/1471-2334-12-80 PMC3340322

[16] De Francesco MA, Caracciolo S, Gargiulo F, Manca N. Phenotypes, genotypes, serotypes and molecular epidemiology of erythromycin-resistant Streptococcus agalactiae in Italy. Eur J Clin Microbiol Infect Dis. 2012;31(8):1741–7. 22120421 10.1007/s10096-011-1495-4

[17] Kong X, Chen T, Guo L, Zhou Y, Lu P, Xiao Y. Phenotypic and genomic comparison of dominant and nondominant sequence-type of Acinetobacter baumannii isolated in China. Front Cell Infect Microbiol. 2023;13:1118285. 36891157 10.3389/fcimb.2023.1118285 PMC9986592

[18] Niu T, Guo L, Kong X, He F, Ru C, Xiao Y. Prevalent Dominant Acinetobacter baumannii ST191/195/208 strains in bloodstream infections have high Drug Resistance and Mortality. Infect Drug Resist. 2023;16:2417–27. 37138832 10.2147/IDR.S403604 PMC10149779

[19] Zarrilli R, Bagattini M, Migliaccio A, Esposito EP, Triassi M. Molecular epidemiology of carbapenem-resistant Acinetobacter baumannii in Italy. Ann Ig. 2021;33(5):401–9. 33270079 10.7416/ai.2020.2395

[20] Oikonomou O, Sarrou S, Papagiannitsis CC, Georgiadou S, Mantzarlis K, Zakynthinos E, et al. Rapid dissemination of colistin and carbapenem resistant Acinetobacter baumannii in Central Greece: mechanisms of resistance, molecular identification and epidemiological data. BMC Infect Dis. 2015;15:559. 26653099 10.1186/s12879-015-1297-x PMC4675053

[21] McKay SL, Vlachos N, Daniels JB, Albrecht VS, Stevens VA, Rasheed JK, et al. Molecular Epidemiology of Carbapenem-Resistant Acinetobacter baumannii in the United States, 2013–2017. Microb Drug Resist. 2022;28(6):645–53. 35639112 10.1089/mdr.2021.0352 PMC9197948

[22] Frenk S, Temkin E, Lurie-Weinberger MN, Keren-Paz A, Rov R, Rakovitsky N, et al. Large-scale WGS of carbapenem-resistant Acinetobacter baumannii isolates reveals patterns of dissemination of ST clades associated with antibiotic resistance. J Antimicrob Chemother. 2022;77(4):934–43. 35084023 10.1093/jac/dkac010

[23] Kim SE, Choi SM, Yu Y, Shin SU, Oh TH, Kang SJ, et al. Replacement of the Dominant ST191 clone by ST369 among Carbapenem-Resistant Acinetobacter baumannii Bloodstream isolates at a Tertiary Care Hospital in South Korea. Front Microbiol. 2022;13:949060. 35910596 10.3389/fmicb.2022.949060 PMC9335038

[24] Ning NZ, Liu X, Bao CM, Chen SM, Cui EB, Zhang JL, et al. Molecular epidemiology of bla (OXA-23) -producing carbapenem-resistant Acinetobacter baumannii in a single institution over a 65-month period in north China. BMC Infect Dis. 2017;17(1):14. 28056839 10.1186/s12879-016-2110-1 PMC5217423

[25] Bankevich A, Nurk S, Antipov D, Gurevich AA, Dvorkin M, Kulikov AS, et al. SPAdes: a new genome assembly algorithm and its applications to single-cell sequencing. J Comput Biol. 2012;19(5):455–77. 22506599 10.1089/cmb.2012.0021 PMC3342519

[26] Seemann T. Prokka: rapid prokaryotic genome annotation. Bioinformatics. 2014;30(14):2068–9. 24642063 10.1093/bioinformatics/btu153

[27] Price MN, Dehal PS, Arkin AP. FastTree 2–approximately maximum-likelihood trees for large alignments. PLoS ONE. 2010;5(3):e9490. 20224823 10.1371/journal.pone.0009490 PMC2835736

[28] Zhou H, Zhang T, Yu D, Pi B, Yang Q, Zhou J, et al. Genomic analysis of the multidrug-resistant Acinetobacter baumannii strain MDR-ZJ06 widely spread in China. Antimicrob Agents Chemother. 2011;55(10):4506–12. 21788470 10.1128/AAC.01134-10 PMC3187012

[29] Letunic I, Bork P. Interactive tree of life (iTOL) v5: an online tool for phylogenetic tree display and annotation. Nucleic Acids Res. 2021;49(W1):W293–6. 33885785 10.1093/nar/gkab301 PMC8265157

[30] Ribeiro-Gonçalves B, Francisco AP, Vaz C, Ramirez M, Carriço JA. PHYLOViZ Online: web-based tool for visualization, phylogenetic inference, analysis and sharing of minimum spanning trees. Nucleic Acids Res. 2016;44(W1):W246–51. 27131357 10.1093/nar/gkw359 PMC4987911

[31] Florensa AF, Kaas RS, Clausen P, Aytan-Aktug D, Aarestrup FM. ResFinder - an open online resource for identification of antimicrobial resistance genes in next-generation sequencing data and prediction of phenotypes from genotypes. Microb Genom. 2022;8(1). 10.1099/mgen.0.000748 PMC8914360 35072601

[32] Wang M, Goh YX, Tai C, Wang H, Deng Z, Ou HY. VRprofile2: detection of antibiotic resistance-associated mobilome in bacterial pathogens. Nucleic Acids Res. 2022;50(W1):W768–73. 35524563 10.1093/nar/gkac321 PMC9252795

[33] Darling AC, Mau B, Blattner FR, Perna NT. Mauve: multiple alignment of conserved genomic sequence with rearrangements. Genome Res. 2004;14(7):1394–403. 15231754 10.1101/gr.2289704 PMC442156

[34] Alikhan NF, Petty NK, Ben Zakour NL, Beatson SA. BLAST Ring Image Generator (BRIG): simple prokaryote genome comparisons. BMC Genomics. 2011;12:402. 21824423 10.1186/1471-2164-12-402 PMC3163573

[35] Siguier P, Perochon J, Lestrade L, Mahillon J, Chandler M. ISfinder: the reference centre for bacterial insertion sequences. Nucleic Acids Res. 2006;34(Database issue):D32–6. 16381877 10.1093/nar/gkj014 PMC1347377

[36] Hu F, Yuan L, Yang Y, Xu Y, Huang Y, Hu Y, et al. A multicenter investigation of 2,773 cases of bloodstream infections based on China antimicrobial surveillance network (CHINET). Front Cell Infect Microbiol. 2022;12:1075185. 36590586 10.3389/fcimb.2022.1075185 PMC9798236

[37] Jiang M, Chen X, Liu S, Zhang Z, Li N, Dong C, et al. Epidemiological analysis of Multidrug-Resistant Acinetobacter baumannii isolates in a Tertiary Hospital over a 12-Year period in China. Front Public Health. 2021;9:707435. 34458227 10.3389/fpubh.2021.707435 PMC8388840

[38] Pournaras S, Koumaki V, Gennimata V, Kouskouni E, Tsakris A. In Vitro Activity of Tigecycline against Acinetobacter baumannii: Global Epidemiology and resistance mechanisms. Adv Exp Med Biol. 2016;897:1–14. 26563303 10.1007/5584_2015_5001

[39] Al-Sweih NA, Al-Hubail MA, Rotimi VO. Emergence of tigecycline and colistin resistance in Acinetobacter species isolated from patients in Kuwait hospitals. J Chemother. 2011;23(1):13–6. 21482488 10.1179/joc.2011.23.1.13

[40] Sun C, Yu Y, Hua X. Resistance mechanisms of tigecycline in Acinetobacter baumannii. Front Cell Infect Microbiol. 2023;13:1141490. 37228666 10.3389/fcimb.2023.1141490 PMC10203620

[41] Guo Y, Han R, Jiang B, Ding L, Yang F, Zheng B, et al. In Vitro Activity of New β-Lactam-β-Lactamase inhibitor combinations and comparators against clinical isolates of Gram-negative Bacilli: results from the China Antimicrobial Surveillance Network (CHINET) in 2019. Microbiol Spectr. 2022;10(4):e0185422. 35862963 10.1128/spectrum.01854-22 PMC9431184

[42] Zhu L, Yan Z, Zhang Z, Zhou Q, Zhou J, Wakeland EK, et al. Complete genome analysis of three Acinetobacter baumannii clinical isolates in China for insight into the diversification of drug resistance elements. PLoS ONE. 2013;8(6):e66584. 23826102 10.1371/journal.pone.0066584 PMC3691203

[43] Shi X, Wang H, Wang X, Jing H, Duan R, Qin S, et al. Molecular characterization and antibiotic resistance of Acinetobacter baumannii in cerebrospinal fluid and blood. PLoS ONE. 2021;16(2):e0247418. 33617547 10.1371/journal.pone.0247418 PMC7899338

[44] You Q, Du X, Hu N, Zhang Y, Zhang N, Wang F, et al. Local characteristics of molecular epidemiolgy of Acinetobacter baumannii in Jilin province (northeast China). BMC Microbiol. 2023;23(1):19. 36658486 10.1186/s12866-023-02761-9 PMC9850558

[45] Ibrahim S, Al-Saryi N, Al-Kadmy IMS, Aziz SN. Multidrug-resistant Acinetobacter baumannii as an emerging concern in hospitals. Mol Biol Rep. 2021;48(10):6987–98. 34460060 10.1007/s11033-021-06690-6 PMC8403534

[46] Bharathi SV, Venkataramaiah M, Rajamohan G. Genotypic and phenotypic characterization of Novel sequence types of Carbapenem-Resistant Acinetobacter baumannii, with heterogeneous resistance determinants and targeted variations in Efflux Operons. Front Microbiol. 2021;12:738371. 35002996 10.3389/fmicb.2021.738371 PMC8735875

[47] Lv W, Zhang X, Hou M, Han D, Li Y, Xiong W. Draft genome sequence of an OXA-23, OXA-66, ADC-25 and TEM-1D co-producing Acinetobacter baumannii ST195 isolated from a patient with neonatal pneumonia in China. J Glob Antimicrob Resist. 2019;16:1–3. 30445210 10.1016/j.jgar.2018.11.008

[48] Liu LL, Ji SJ, Ruan Z, Fu Y, Fu YQ, Wang YF, et al. Dissemination of blaOXA-23 in Acinetobacter spp. in China: main roles of conjugative plasmid pAZJ221 and transposon Tn2009. Antimicrob Agents Chemother. 2015;59(4):1998–2005. 25605357 10.1128/AAC.04574-14 PMC4356780

[49] Hu Y, Zhang X, Deng S, Yue C, Jia X, Lyu Y. Non-antibiotic prevention and treatment against Acinetobacter baumannii infection: are vaccines and adjuvants effective strategies? Front Microbiol. 2023;14:1049917. 36760499 10.3389/fmicb.2023.1049917 PMC9905804

[50] Yoon EJ, Kim JO, Yang JW, Kim HS, Lee KJ, Jeong SH, et al. The blaOXA-23-associated transposons in the genome of Acinetobacter spp. represent an epidemiological situation of the species encountering carbapenems. J Antimicrob Chemother. 2017;72(10):2708–14. 29091183 10.1093/jac/dkx205

